# Supplementary material for: Optimization of seebeck coefficients in polyaniline-doped manganese dioxide nanocomposites
Source: PLoS One. 2025 Apr 16;20(4):e0321385. doi: 10.1371/journal.pone.0321385 (PMC12002486; doi:10.1371/journal.pone.0321385)
Supplement: S1 File — (DOCX) [file pone.0321385.s001.docx]

# Supplementary Information: Seebeck Measuring Instrument and Data Analysis

Jay Molino ^a,b^, Muhammad Ibrahim ^c^, Rolando Serra ^d^, Svetlana de Tristán^a^

^a^Universidad Especializada de las Américas (UDELAS), Faculty of Biosciences and Public Health, Biomedical Engineering, Centro I+D+i de Biotecnología, Energías Verdes y Cambio Climático, Albrook, Paseo de La Iguana 0843-014, Republic of Panama.

^b^Sistema Nacional de Investigación (SNI), SENACYT, Panama City, Republic of Panama

*^c^Faculty of Engineering and Science, Bahauddin Zakariya University – BZU, Punjab, Pakistan.*

*^d^Departamento de Física, Universidad Tecnológica de La Habana José Antonio Echeverría, Cuba.*

### Seebeck Measuring Instrument and measurements

Thin films of HCl-doped polyaniline (PANI) and its composites were prepared using the doctor-blade method. The Seebeck measuring setup consisted of the following components:

- **Hot Plate**: Created a controlled temperature gradient across the sample.
- **Nano-Voltmeter (2182A KEITHELY)**: Measured the voltage difference generated due to the temperature gradient.
- **Thermocouples**: Measured the temperature at different points on the sample.
- **Sample Holder**: Maintained a constant distance of 2 cm between the contact points.
- **Temperature Range**: Measurements were conducted from 313K to 373K.

$$S=\frac{\Delta V}{\Delta T}$$

where ΔV is the thermoelectric potential difference and ΔT is the temperature gradient across the material.

### D.C. Conductivity Measurements

The D.C. electrical conductivity of all prepared samples was measured using a two-probe method, applying a voltage range of 20V. Conducting polymers, when doped, exhibit a range of electrical conductivities from semiconducting to metallic. The conductivity of these materials also depends on temperature, generally increasing with rising temperature, indicative of semiconducting behavior. Polyaniline (PANI), a prominent conducting polymer, demonstrates this temperature-dependent conductivity. Table 1, table 2 and 3 summarize the D.C. conductivity data for different samples at various temperatures, the seebeck coefficient for different samples and the activation energy data for different samples are summarized in Table 3 respectively.

Table 1. Conductivity for different samples at different temperatures.

| **Temperature (K)** | **Pure PANI (S/cm)** | **PANI-HCl (S/cm)** | **PANI-5% MnO_2_ (S/cm)** | **PANI-15% MnO_2_ (S/cm)** |
| --- | --- | --- | --- | --- |
| 313 | 8.51 × 10⁻⁷ | 9.75 × 10⁻⁶ | 1.05 × 10⁻⁵ | 1.15 × 10⁻⁵ |
| 333 | 1.20 × 10⁻⁶ | 2.40 × 10⁻⁵ | 3.15 × 10⁻⁵ | 3.35 × 10⁻⁵ |
| 353 | 2.80 × 10⁻⁶ | 5.85 × 10⁻⁵ | 7.05 × 10⁻⁵ | 7.45 × 10⁻⁵ |
| 373 | 6.30 × 10⁻⁶ | 1.25 × 10⁻⁴ | 1.55 × 10⁻⁴ | 1.65 × 10⁻⁴ |
| 393 | 2.25 × 10⁻⁴ | 6.45 × 10⁻⁴ | 9.03 × 10⁻⁴ | 9.53 × 10⁻⁴ |

**Table 2. Seebeck Coefficient of Various Samples at Different Temperatures**

| **Temperature (K)** | **Pure PANI (mV/K)** | **PANI-HCl (mV/K)** | **PANI-5% MnO2 (mV/K)** | **PANI-15% MnO2 (mV/K)** |
| --- | --- | --- | --- | --- |
| 313 | 0 | 10 | 20 | 25 |
| 333 | 5 | 15 | 25 | 30 |
| 353 | 10 | 20 | 30 | 35 |
| 373 | 15 | 25 | 35 | 40 |
| 393 | 20 | 30 | 40 | 45 |

**Table 3. Activation Energy for Various Samples Activation Energy for PANI-Doped with HCl and PANI-MnO_2_ Composites**

| **Sample** | **Slope (m)** | **Activation Energy (eV)** |
| --- | --- | --- |
| PANI-HCl | -9505 | 0.818 |
| PANI-HCl-5% MnO2 | -7364 | 0.634 |
| PANI-HCl-15% MnO2 | -5904 | 0.508 |

### Current Density Measurements

Table 4 shows that the current density increases with temperature and MnO2 content.. The current density of HCl-doped polyaniline and PANI with HCl-MnO2 composite can be calculated using the following relation:

J=σ(E_emf_−ΔV) and J=σ(E_emf_​ − SΔT)

**Table 4. Current Density of PANI-Doped with HCl and HCl-MnO2 Composite at Different Temperatures**

| **Temperature (K)** | **Current Density of PANI Doped with HCl (mA/cm²)** | **Current Density of PANI Doped with HCl-MnO2 Composite (mA/cm²)** |
| --- | --- | --- |
| 313 K | 0 mA/cm² | 0.122 mA/cm² |
| 333 K | 0.395 mA/cm² | 2.368 mA/cm² |
| 353 K | 1.17 mA/cm² | 8.58 mA/cm² |
| 373 K | 3.57 mA/cm² | 17.57 mA/cm² |

Figure S1 indicates that the current density (J) of polyaniline doped with HCl-MnO2 composites increases more rapidly than polyaniline doped with HCl alone. Increasing the temperature and wt% of MnO2 enhances the efficiency of charge carriers, resulting in higher Seebeck voltage and increased current density.


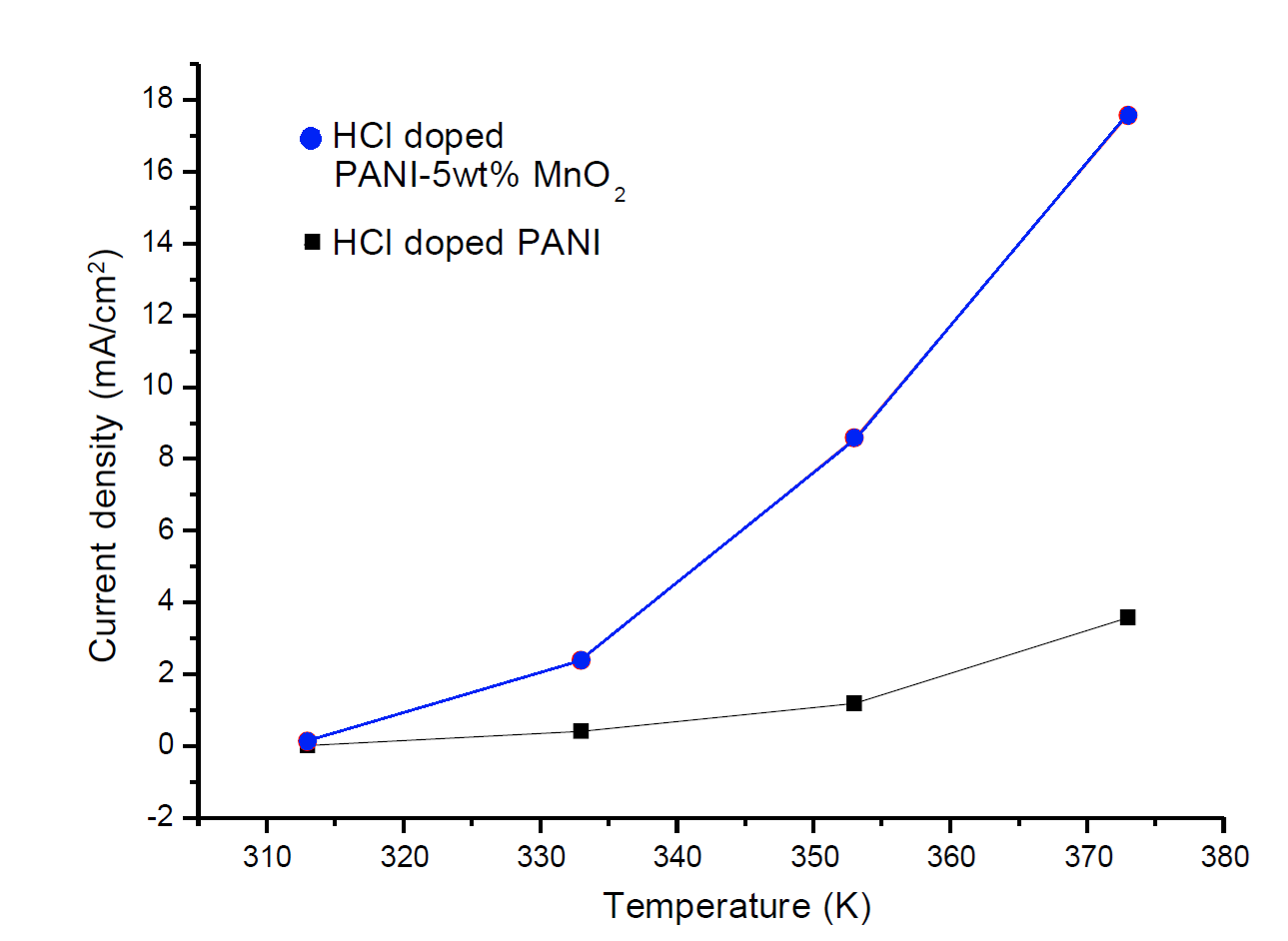


**Current density(J) of PANI-doped with HCl and PANI with HCl-5wt%** 𝐌𝐧𝐎𝟐

Notice that the detailed results from the XRD analysis confirm the successful synthesis and incorporation of MnO_2_ into the polyaniline matrix, enhancing the structural and crystalline properties of the composites. The observed peaks are consistent with the reported values in the literature, validating the synthesis process and the composite's crystalline nature. The D.C. electrical conductivity measurements demonstrated that the conductivity of the composites increases with both temperature and MnO_2_ content. The activation energy decreased with higher MnO_2_ content, indicating improved charge carrier mobility. The Seebeck coefficient measurements showed significant increases with temperature and MnO_2_ content, confirming the enhanced thermoelectric properties of the composites.
