# Supplementary material for: Optimization of seebeck coefficients in polyaniline-doped manganese dioxide nanocomposites
Source: PLoS One. 2025 Apr 16;20(4):e0321385. doi: 10.1371/journal.pone.0321385 (PMC12002486; doi:10.1371/journal.pone.0321385)
Supplement: Attachment — Submitted filename: PONE-D-24-32705_reviewer.pdf [file pone.0321385.s003.pdf]

PLOS ONE

Seebeck Coefficients Optimization in Doped Polyaniline-Manganese Dioxide Nanocomposites

--Manuscript Draft--

**Manuscript Number:**	PONE-D-24-32705
**Article Type:**	Research Article
**Full Title:**	Seebeck Coefficients Optimization in Doped Polyaniline-Manganese Dioxide Nanocomposites
**Short Title:**	Optimizing Seebeck Coefficients in Doped PANI-MnO2 Nanocomposites
**Corresponding Author:**	Jay Jesus Molino, Ph.D.<br>Universidad Especializada de las Americas<br>Panama, Panama PANAMA
**Keywords:**	Seebeck coefficient, Polyaniline (PANI), Manganese dioxide (MnO2), Thermoelectric properties, Conducting polymers, Nanocomposites, Electrical conductivity
**Abstract:**	Polyaniline (PANI) and PANI-MnO2 composites were synthesized via a chemical route with varying manganese dioxide (MnO2) content, specifically 5wt% and 15wt%. X-ray diffraction (XRD) confirmed the structural formation of both PANI and PANI-MnO2 composites. The direct current conductivity was measured, showing an increase with temperature: at 393K, pure PANI had a conductivity of 2.25 × 10-4 S/cm, which increased significantly in the composites, reaching 9.03 × 10-4 S/cm for the 15wt% MnO2 composite. The Seebeck coefficient also increased with temperature and MnO2 concentration, achieving a maximum value of 52 mV K-1 at 373K for the 15wt% MnO2 composite. These results indicate that the synthesized PANI- MnO2 composites exhibit semiconducting behavior with improved thermoelectric properties, making them promising candidates for applications in thermoelectric devices such as generators and thermopiles. The study highlights the potential of these materials in enhancing the efficiency of thermoelectric energy conversion.
**Order of Authors:**	Jay Jesus Molino, Ph.D.
	Muhammad Ibrahim
	Rolando Serra
	Svetlana de Tristán
**Additional Information:**	
**Question**	**Response**
**Financial Disclosure**<br><br>Enter a financial disclosure statement that describes the sources of funding for the work included in this submission. Review the submission guidelines for detailed requirements. View published research articles from *PLOS ONE* for specific examples.<br><br>This statement is required for submission and **will appear in the published article** if the submission is accepted. Please make sure it is accurate.	The author(s) received no specific funding for this work.

Powered by Editorial Manager® and ProduXion Manager® from Aries Systems Corporation

**Funded studies**<br>Enter a statement with the following details:<br>• Initials of the authors who received each award<br>• Grant numbers awarded to each author<br>• The full name of each funder<br>• URL of each funder website<br>• Did the sponsors or funders play any role in the study design, data collection and analysis, decision to publish, or preparation of the manuscript?<br><br>Did you receive funding for this work?	
**Competing Interests**<br><br>Use the instructions below to enter a competing interest statement for this submission. On behalf of all authors, disclose any competing interests that could be perceived to bias this work—acknowledging all financial support and any other relevant financial or non-financial competing interests.<br><br>This statement is required for submission and **will appear in the published article** if the submission is accepted. Please make sure it is accurate and that any funding sources listed in your Funding Information later in the submission form are also declared in your Financial Disclosure statement.<br><br>View published research articles from *PLOS ONE* for specific examples.	The authors have declared that no competing interests exist.

Powered by Editorial Manager® and ProduXion Manager® from Aries Systems Corporation

**NO authors have competing interests**<br><br>Enter: *The authors have declared that no competing interests exist.*<br><br>**Authors with competing interests**<br><br>Enter competing interest details beginning with this statement:<br><br>*I have read the journal's policy and the authors of this manuscript have the following competing interests: [insert competing interests here]*<br><br>* typeset	
**Ethics Statement**<br><br>Enter an ethics statement for this submission. This statement is required if the study involved:<br><br>• Human participants<br>• Human specimens or tissue<br>• Vertebrate animals or cephalopods<br>• Vertebrate embryos or tissues<br>• Field research<br><br>Write "N/A" if the submission does not require an ethics statement.<br><br>General guidance is provided below. Consult the submission guidelines for detailed instructions. **Make sure that all information entered here is included in the Methods section of the manuscript.**	NA

**Format for specific study types**<br><br>**Human Subject Research (involving human participants and/or tissue)**<br>• Give the name of the institutional review board or ethics committee that approved the study<br>• Include the approval number and/or a statement indicating approval of this research<br>• Indicate the form of consent obtained (written/oral) or the reason that consent was not obtained (e.g. the data were analyzed anonymously)<br><br>**Animal Research (involving vertebrate animals, embryos or tissues)**<br>• Provide the name of the Institutional Animal Care and Use Committee (IACUC) or other relevant ethics board that reviewed the study protocol, and indicate whether they approved this research or granted a formal waiver of ethical approval<br>• Include an approval number if one was obtained<br>• If the study involved *non-human primates*, add *additional details* about animal welfare and steps taken to ameliorate suffering<br>• If anesthesia, euthanasia, or any kind of animal sacrifice is part of the study, include briefly which substances and/or methods were applied<br><br>**Field Research**<br><br>Include the following details if this study involves the collection of plant, animal, or other materials from a natural setting:<br>• Field permit number<br>• Name of the institution or relevant body that granted permission	
**Data Availability**<br><br>Authors are required to make all data underlying the findings described fully available, without restriction, and from the time of publication. PLOS allows rare exceptions to address legal and ethical concerns. See the PLOS Data Policy and FAQ for detailed information.	Yes - all data are fully available without restriction

Powered by Editorial Manager® and ProduXion Manager® from Aries Systems Corporation

A Data Availability Statement describing where the data can be found is required at submission. Your answers to this question constitute the Data Availability Statement and **will be published in the article**, if accepted.<br><br>**Important:** Stating 'data available on request from the author' is not sufficient. If your data are only available upon request, select 'No' for the first question and explain your exceptional situation in the text box.<br><br>Do the authors confirm that all data underlying the findings described in their manuscript are fully available without restriction?	
**Describe where the data may be found in full sentences. If you are copying our sample text, replace any instances of XXX with the appropriate details.**<br><br>• If the data are **held or will be held in a public repository**, include URLs, accession numbers or DOIs. If this information will only be available after acceptance, indicate this by ticking the box below. For example: *All XXX files are available from the XXX database (accession number(s) XXX, XXX.).*<br>• If the data are all contained **within the manuscript and/or Supporting Information files**, enter the following: *All relevant data are within the manuscript and its Supporting Information files.*<br>• If neither of these applies but you are able to provide **details of access elsewhere**, with or without limitations, please do so. For example:<br><br>*Data cannot be shared publicly because of [XXX]. Data are available from the XXX Institutional Data Access / Ethics Committee (contact via XXX) for researchers who meet the criteria for access to confidential data.*<br><br>*The data underlying the results presented in the study are available from (include the name of the third party*	All the data can be found in the main text and supplemental material.

and contact information or URL).<br>• This text is appropriate if the data are owned by a third party and authors do not have permission to share the data.<br><br>* typeset	
Additional data availability information:	

Powered by Editorial Manager® and ProduXion Manager® from Aries Systems Corporation

Manuscript

Click here to access/download;Manuscript;SeebeckCoefficients_01082024.do

Seebeck Coefficients Optimization in Doped Polyaniline-Manganese Dioxide Nanocomposites

Jay Molino [a,b], Muhammad Ibrahim [c], Rolando Serra [d], Svetlana de Tristán[a]

[a]Laboratorio de Investigación Experimental de Bioseñales, Centro I+D+i de Biotecnología, Energías Verdes y Cambio Climático, Universidad Especializada de las Américas (UDELAS), Albrook, Paseo de La Iguana 0843-014, Republic of Panama.
[b]Sistema Nacional de Investigación (SNI), SENACYT, Panama City, Republic of Panama
[c]Faculty of Engineering and Science, Bahauddin Zakariya University – BZU, Punjab, Pakistan.
[d]Departamento de Física, Universidad Tecnológica de La Habana José Antonio Echeverría, Cuba.

Abstract

Polyaniline (PANI) and PANI-MnO_2 composites were synthesized via a chemical route with varying manganese dioxide (MnO_2) content, specifically 5wt% and 15wt%. X-ray diffraction (XRD) confirmed the structural formation of both PANI and PANI-MnO_2 composites. The direct current conductivity was measured, showing an increase with temperature: at 393K, pure PANI had a conductivity of 2.25×10^{-4} S/cm, which increased significantly in the composites, reaching 9.03×10^{-4} S/cm for the 15wt% MnO_2 composite. The Seebeck coefficient also increased with temperature and MnO_2 concentration, achieving a maximum value of 52 mV K^{-1} at 373K for the 15wt% MnO_2 composite. These results indicate that the synthesized PANI- MnO_2 composites exhibit semiconducting behavior with improved thermoelectric properties, making them promising candidates for applications in thermoelectric devices such as generators and thermopiles. The study highlights the potential of these materials in enhancing the efficiency of thermoelectric energy conversion.

Keywords: Seebeck coefficient, Polyaniline (PANI), Manganese dioxide (MnO2), Thermoelectric properties, Conducting polymers, Nanocomposites, Electrical conductivity

Introduction

The study of thermoelectric materials has gained significant traction due to their potential to convert waste heat into useful electrical energy, addressing global energy concerns and contributing to sustainability (Rowe, 1995; Tritt, 2001). Thermoelectric devices rely on the Seebeck Effect and require materials with high Seebeck coefficients, excellent electrical conductivity, and low thermal conductivity for efficient energy conversion (Snyder & Toberer, 2008). Conducting polymers, such as polyaniline (PANI), have emerged as promising candidates for thermoelectric applications due to their tunable electrical properties, ease of synthesis, and environmental stability (Ramanathan, 2012; Zhou et al., 2011).

Polyaniline (PANI) is a conducting polymer known for its unique electrical properties and ability to exist in various oxidation states, controllable through doping (MacDiarmid et al., 1985; Chiang & MacDiarmid, 1986). The emeraldine form of PANI exhibits significant conductivity when

doped with protonic acids, making it a versatile material for electronic applications such as sensors, batteries, and thermoelectric devices (Jang et al., 2014; Lu et al., 2018).

Manganese dioxide (MnO_2), an inorganic compound with a high Seebeck coefficient, has been widely studied for its potential in energy storage and conversion devices (Whittingham, 2004; Bruce et al., 2008). MnO_2 has several polymorphic forms, each with distinct structural and electrochemical properties (Post, 1999). Combining MnO_2 with PANI can enhance the thermoelectric properties of the composite material, leveraging the high Seebeck coefficient of MnO_2 and the conductive nature of PANI (Zhou et al., 2010; Zhao et al., 2019).

This study synthesized PANI and PANI- MnO_2 composites with varying MnO_2 content (5wt% and 15wt%) using a chemical route. The structural properties of these composites were confirmed using X-ray diffraction (XRD). The D.C. conductivity was measured as a function of temperature, and the Seebeck coefficient was evaluated to determine the thermoelectric potential of the composites. This research aims to optimize the Seebeck coefficient in PANI-MnO_2 nanocomposites and assess their suitability for thermoelectric applications.

Doping PANI with different materials, such as MnO_2, can significantly influence its electrical conductivity and thermoelectric properties (Chen et al., 2015). By optimizing doping levels and composite structures, it is possible to enhance the Seebeck coefficient, making PANI-MnO_2 composites highly efficient for thermoelectric applications, which involves maintaining high electrical conductivity while minimizing thermal conductivity, thus maximizing thermoelectric efficiency (Kim et al., 2013).

This research focuses on developing and characterizing conducting polymers and transition metal oxides, exploring new avenues for their application in thermoelectric devices. By enhancing the thermoelectric performance of PANI through MnO_2 doping, we aim to develop efficient, environmentally friendly materials for energy conversion technologies. The findings of this study have the potential to impact the design and optimization of next-generation thermoelectric devices, which are critical for improving energy efficiency in various industrial processes (Shakouri, 2011; Dresselhaus et al., 2007).

Materials and Methods

PANI was synthesized by mixing ammonium persulfate (APS) with aniline in an acidic medium, followed by filtration, washing, drying, and grinding. HCl-doped PANI and PANI- MnO_2 composites (5% and 15% MnO_2) were prepared using a similar method, adjusting MnO_2 concentrations. XRD confirmed structural properties, and via SEM we analyzed the morphology. Electrical properties were measured using a Keithley 2400 SourceMeter. Seebeck coefficients were determined using a custom setup with a nano-voltmeter, measuring from 313K to 373K.

Synthesis of Pure PANI

Polyaniline (PANI) was synthesized by dissolving 12.25 g of APS in 50 mL of distilled water, stirring continuously. Separately, 50 mL of deionized water was mixed with 5 mL of aniline for 30 minutes. The APS solution was added dropwise to the aniline solution, followed by 5 mL of hydrochloric acid (HCl), maintaining a neutral pH. The reaction mixture was stirred for 30-60 minutes and left in the dark for 24 hours. The resulting PANI precipitate was filtered, washed with deionized water, dried at 60°C, and ground into a fine powder.

Synthesis of HCl-Doped PANI

HCl-doped PANI was prepared by dissolving 12.25 g of APS in 50 mL of HCl and stirring. Separately, 50 mL of HCl was mixed with 5 mL of aniline for 30 minutes. The APS solution was added dropwise to the aniline solution, followed by 5 mL of HCl, maintaining a neutral pH. The mixture was stirred for 30-60 minutes and left in the dark for 24 hours. The resulting precipitate was filtered, washed with deionized water, dried at 60°C, and ground into a fine powder.

Synthesis of HCl-Doped Polyaniline- MnO_2 Composites

HCl-doped PANI- MnO_2 composites were synthesized by preparing two APS solutions in 50 mL of HCl with 12.25 g of APS. For the 5% MnO2 composite, 0.25 g of MnO_2 was added to 50 mL of HCl, stirred for 30 minutes, followed by 5 mL of aniline. The mixture was combined with the APS solution, stirred, left in the dark for 24 hours, filtered, dried at 90°C, and ground. The same procedure was followed for the 15% MnO_2 composite, using 0.75 g of MnO_2.

Characterization

Structural properties were analyzed using a Bruker D8 Advance diffractometer with Cu Kα radiation (λ = 1.5406 Å), scanning from 10° to 80° at a rate of 2° per minute. After gold-coating the samples, surface morphology and microstructure were examined using a JEOL JSM-6490LV microscope. Conductivity was measured using a Keithley 2400 SourceMeter in the temperature range of 313K to 393K with a two-probe method. The Seebeck coefficient was measured using a custom setup with a temperature control system and a nano-voltmeter (2182A KEITHLEY). Thin films for the Seebeck coefficient measurements were prepared by the doctor-blade method, and measurements were taken from 313K to 373K with a 2 cm distance between contact points.

Results

X-ray Diffraction (XRD) Analysis

The XRD patterns for pure polyaniline, HCl-doped polyaniline, HCl-doped polyaniline with 5wt% MnO_2, and HCl-doped polyaniline with 15wt% MnO_2 are presented in Figure 1. The XRD pattern for pure polyaniline (PANI) exhibits crystalline peaks ranging from 2θ = 9.5° to 2θ = 29.9°, with prominent peaks at 2θ = 20.77° and 26.63° corresponding to the (020) and (200) crystallographic planes, respectively, indicating its amorphous nature. The broad peak at 26.63° is consistent with reported data, demonstrating the characteristic broad peak of polyaniline (Figure 1-A).

The XRD pattern also shows partially crystalline peaks for HCl-doped polyaniline compared to pure PANI. Broad peaks at 2θ = 14.57° and 20.22° and a semi-crystalline peak at 2θ = 25.23° with a d-value of 2.98 Å indicate a slight crystalline nature due to π-conjugation in polyaniline (Figure 1-B).

Comparing the XRD patterns of pure polyaniline and HCl-doped polyaniline reveals a hump peak at 2θ = 25.23° in the HCl-doped sample, indicating a partially crystalline nature. This semi-crystalline arrangement suggests an interaction mechanism between the polymer particles and the dopant (Figure 1-C). The XRD pattern of pure MnO_2 shows well-defined peaks at angles 2θ = 22.29°, 37.2°, 42.2°, 55.3°, and 66.63°, corresponding to the (120), (131), (300), (160), and (421) planes, respectively. The high-intensity peak at 2θ = 37.2° with a d-value of 2.38 Å confirms the crystalline nature of MnO_2. The average grain size, calculated using Scherer's formula, was determined to be 55 nm (Figure 1-D).

Which peaks support this statement?

The XRD patterns of HCl-doped polyaniline-MnO_2 composites with 5wt% and 15wt% MnO_2 show partially sharp peaks of MnO_2 integrated with HCl-doped polyaniline. The crystallinity of MnO_2 within the polymer matrix increases with MnO_2 concentration, enhancing the conductive nature and confirming the homogeneous mixing of MnO_2 within the polyaniline chain (Figure 1-E).

The detailed results from the XRD analysis confirm the successful synthesis and incorporation of MnO2 into the polyaniline matrix, enhancing the structural and crystalline properties of the composites. The observed peaks are consistent with the reported values in the literature, validating the synthesis process and the composite's crystalline nature.

Fig 1A and B can be removed

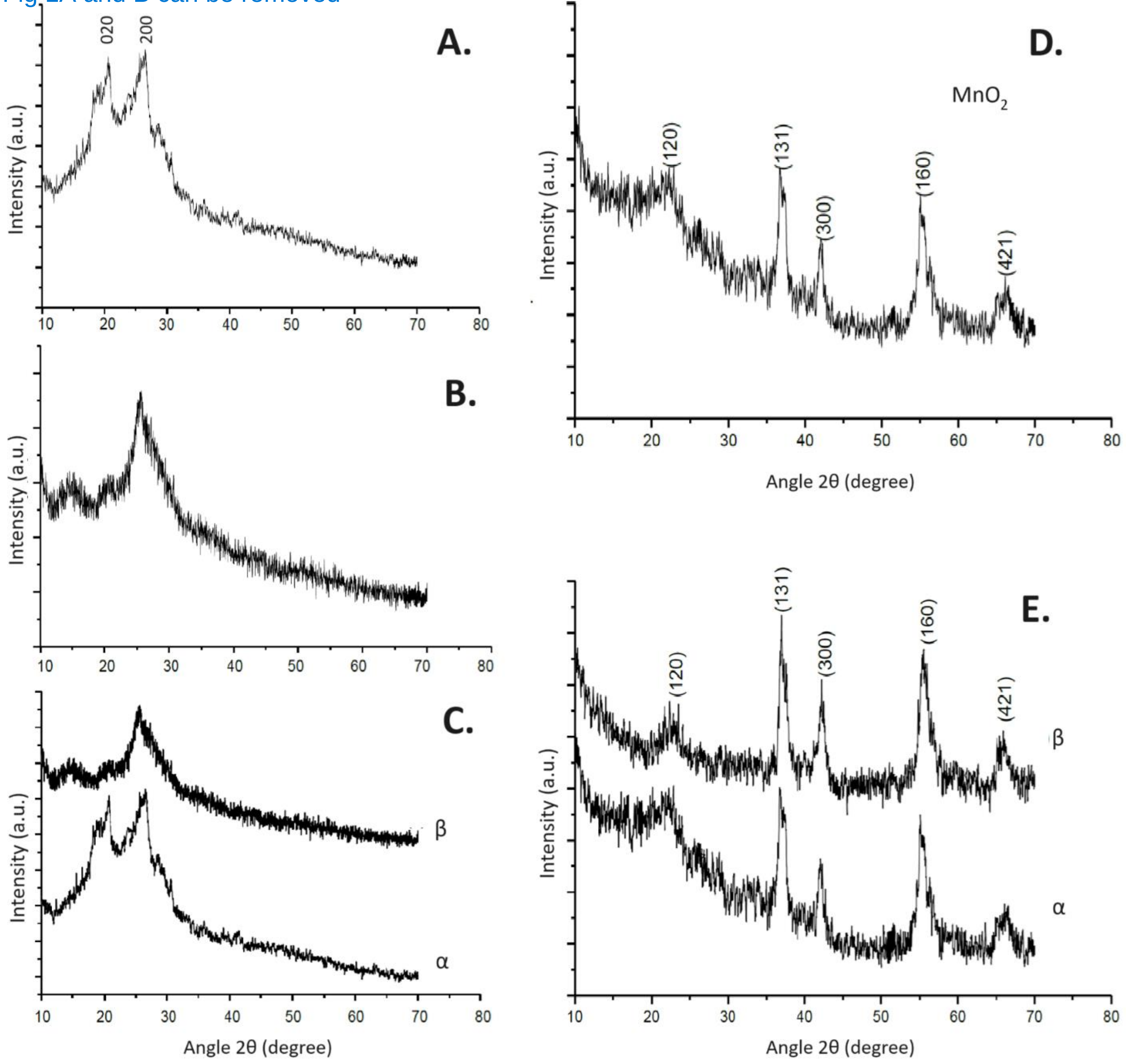

Figure 1: *Panel A*. X-ray diffraction of pure polyaniline; *Panel B*. XRD of Polyaniline doped with HCl; *Panel C*. XRD of (α) Pure polyaniline (β) HCl doped polyaniline; *Panel D*. XRD of MnO2; XRD of (α) HCl doped Polyaniline-5wt% MnO2 (β) HCl doped Polyaniline-15wt% MnO2.

D.C. Electrical Conductivity Measurements

The D.C. electrical conductivity of all prepared samples was measured using a two-probe method, applying a voltage range of 20V. Conducting polymers, when doped, exhibit a range of electrical conductivities from semiconducting to metallic. The conductivity of these materials also depends on temperature, generally increasing with rising temperature, indicative of semiconducting behavior. Polyaniline (PANI), a prominent conducting polymer, demonstrates this temperature-dependent conductivity.

The I-V characteristics of pure PANI were studied at various temperatures (313K-393K). The I-V curves indicate that the resistivity of pure PANI is higher than that of HCl-doped PANI and its composites. Consequently, the D.C. conductivity of pure PANI is lower. As temperature increases, the conductivity of pure PANI also increases (Figure 2-A).

The I-V characteristics of HCl-doped PANI were evaluated at various temperatures (313K-393K). The resistance (R) was calculated using Ohm's law and converted to conductivity (σ) using the equation $\sigma = L/RA$. The resistivity of HCl-doped PANI is lower than that of pure PANI, resulting in higher conductivity, which increases with temperature, as shown in Figure 2-B.

The electrical properties of HCl-doped PANI-5wt% MnO2 were observed at temperatures ranging from 313K to 393K. The I-V curves were analyzed to calculate resistance and conductivity. The resistivity of this composite is lower than that of pure PANI, indicating higher conductivity, as shown in Figure 2-C.

Similarly, the I-V characteristics of HCl-doped PANI-15wt% MnO_2 were measured. The resistivity of this composite is lower than that of pure PANI and decreases with increasing MnO_2 content, resulting in higher conductivity, as depicted in Figure 2-D.

Discuss the data, give values of resistance and conductivity (as obtained by Ohm's law) instead of just discuss on which one is higher/lower. Compare the values obtained by 5wt% and 15wt% MnO2-PANI with HCl-doped PANI (Fig 2B, C and D.

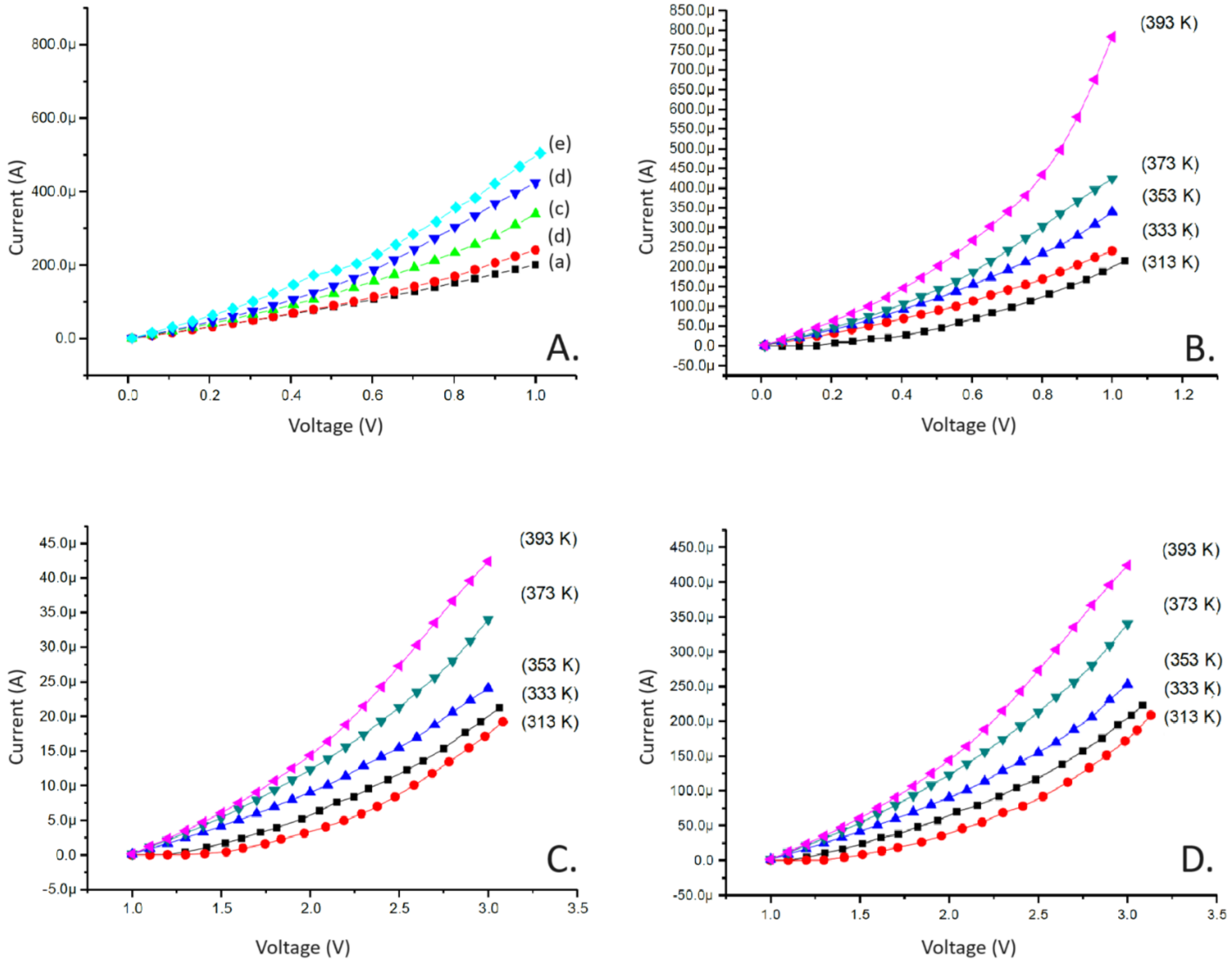

Figure 2: *Panel A*. Characteristic I-V curve of Pure PANI at various temperatures (a) 313K (b) 333K (c) 353K (d) 373K (e) 393K. *Panel B*. Characteristic I-V curve of HCl doped PANI at various temperatures (a) 313K (b) 333K (c) 353K (d) 373K (e) 393K. *Panel C*. Characteristic I-V curve of PANI 5wt%-$\mathbf{MnO_2}$ at various temperatures (a) 313K (b) 333K (c) 353K (d) 373K (e) 393K. *Panel D*. Characteristic I-V curve of PANI 15wt%-$\mathbf{MnO_2}$ at various temperatures (a) 313K (b) 333K (c) 353K (d) 373K (e) 393K

D.C. Conductivity and Seebeck Coefficient Analysis of HCl-Doped PANI and HCl-MnO_2 Composites

The D.C. conductivity of PANI-doped with HCl and its MnO2 composites was studied as a function of temperature. The results show that conductivity increases with temperature and MnO2 content, as illustrated in Figure 3-A. MnO2, an n-type semiconductor with a large band gap, enhances carrier concentration and conductivity.

The activation energy for these composites was calculated using temperature-dependent D.C. conductivity data. By applying a linear fit to ln(σ) vs. 1/T, the activation energy was determined and found to decrease with increasing MnO2 content (Figure 3-B). This reduction in activation energy signifies enhanced charge carrier mobility and reduced energy barriers, which is further evidenced by Figure 13, showing a significant decrease in activation energy with higher MnO2

content. These findings confirm that the HCl-doped PANI-MnO2 composites exhibit improved conductivity with increasing temperature and MnO2 content, consistent with the behavior of semiconducting materials where higher temperatures facilitate better charge carrier movement.

The Seebeck coefficient of HCl-doped PANI also demonstrated a significant increase with temperature. Initially, at 0 mV/K at 313K, the Seebeck coefficient rose to 32 mV/K at 373K, illustrating the semiconducting behavior of HCl-doped PANI, where higher temperatures enhance charge carrier movement (Figure 3-D).

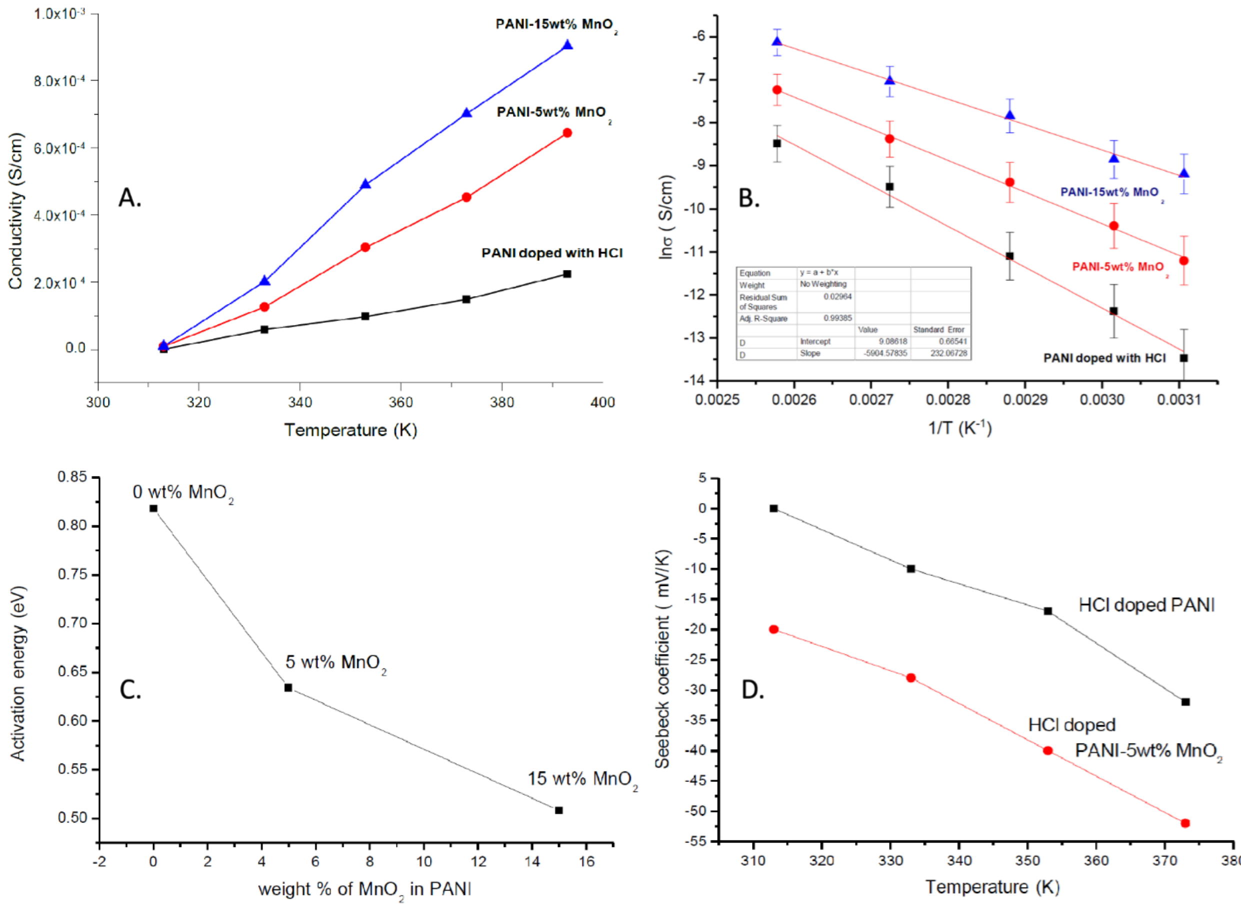

Figure 3. *Panel 1*: D.C conductivity vs temperature dependent for (a) polyaniline doped with HCl (b) polyaniline doped with HCl-5wt% $\mathbf{MnO_2}$ (c) polyaniline doped with HCl-15wt% $\mathbf{MnO_2}$; *Panel 2*: ln(σ) vs 1/T for (a) polyaniline doped with HCl (b) polyaniline doped with HCl-5wt% $\mathbf{MnO_2}$ (c) polyaniline doped with HCl-15wt% $\mathbf{MnO_2}$; Panel 3: plot of Activation energy vs wt% of $\mathbf{MnO_2}$ in PANI; Panel 4: Seebeck coefficient of PANI-doped with HCl and PANI with HCl-5wt% $\mathbf{MnO_2}$

For HCl-doped PANI-MnO2 composites, the Seebeck coefficient increased significantly with the addition of MnO2 nanoparticles. The Seebeck coefficient for the HCl-doped PANI-MnO2 composite increased from 32 mV/K at 313K to 52 mV/K at 373K, indicating enhanced thermoelectric performance due to improved charge carrier efficiency. This improvement is more pronounced in the composites compared to HCl-doped PANI alone, where the Seebeck coefficient ranged from 0 mV/K to 32 mV/K, while for the composites it ranged from 20 mV/K to 52 mV/K

The range of Seebeck coefficient improvement is just the same (32 mV/K) for both. Discuss.

over the temperature range of 313K to 373K. The increase in the Seebeck coefficient with temperature and MnO2 content is attributed to the enhanced efficiency of charge carriers and the generation of more Seebeck voltage.

Discussion

The discussion can be combined in the results section.

The X-ray diffraction (XRD) analysis provided vital insights into the crystalline structure of the synthesized samples. Pure polyaniline (PANI) exhibited broad peaks at 2θ = 20.77° and 26.63°, corresponding to the (020) and (200) crystallographic planes, indicating its amorphous nature. Upon doping with HCl, PANI showed partially crystalline characteristics with peaks at 2θ = 14.57°, 20.22°, and 25.23°. This increase in crystallinity suggests enhanced alignment and ordering of the polymer chains due to HCl doping.

Further addition of MnO_2 to the HCl-doped PANI increased the crystallinity significantly. The XRD patterns of the HCl-doped PANI- MnO_2 composites displayed sharp peaks corresponding to MnO2 at 2θ = 22.29°, 37.2°, 42.2°, 55.3°, and 66.63°, confirming the formation of the composite and its orthorhombic structure. The intensity of these peaks increased with higher MnO_2 content, indicating better dispersion and interaction of MnO_2 within the PANI matrix.

The D.C. electrical conductivity measurements demonstrated an explicit dependency on temperature and dopant concentration. Pure PANI exhibited the lowest conductivity, increasing substantially upon HCl doping. This enhancement in conductivity is attributed to the introduction of charge carriers and improved polymer chain ordering due to HCl doping. Adding MnO_2 to the HCl-doped PANI further increased the conductivity, with the HCl-doped PANI-15 wt% MnO_2 composite showing the highest conductivity; which indicates that MnO_2 effectively enhances the charge transport properties of PANI. The conductivity values ranged from 2.25×10^{-4} S/cm for pure PANI to 9.03×10^{-4} S/cm for the 15 wt% MnO_2 composite at 393K. The significant increase in conductivity is likely due to the synergistic effect of MnO_2 and HCl doping, which improves the electronic structure and reduces the energy barrier for charge transport.

Seebeck coefficient measurements revealed positive values for all samples, which is characteristic of p-type semiconductors. The Seebeck coefficient increased with temperature and MnO_2 content, reflecting improved thermoelectric performance. The highest Seebeck coefficient was observed for the HCl-doped PANI-15 wt% MnO_2 composite, with a value of 52 mV/K at 373K, compared to 32 mV/K for HCl-doped PANI. This enhancement is attributed to the increased carrier concentration and improved mobility within the composite. The presence of MnO_2, with its wide band gap, helps increase the energy of charge carriers above the Fermi level, thereby enhancing the Seebeck coefficient.

The activation energy for electrical conduction decreased with increasing MnO_2 content. Pure PANI had the highest activation energy of 0.818 eV, which decreased to 0.508 eV for the HCl-doped PANI-15 wt% MnO_2 composite. This reduction in activation energy indicates that MnO2 doping facilitates easier charge carrier movement, enhancing electrical conductivity and thermoelectric properties. The decrease in activation energy with higher MnO2 content suggests that MnO2 acts as an efficient dopant, reducing the energy barriers within the polymer matrix and improving the overall charge transport properties.

Conclusion

This study successfully utilized the in-situ molecular polymerization approach to manufacture pure polyaniline (PANI), HCl-doped PANI, and HCl-doped PANI-MnO2 composites. While HCl-doped PANI-MnO2 has been previously reported, comprehensive studies on its temperature-dependent D.C. conductivity and Seebeck coefficient were notably absent.

X-Ray diffraction patterns confirmed that manganese dioxide nanoparticles are partially crystalline, whereas pure PANI exhibits an amorphous nature. The XRD patterns of HCl-doped PANI-MnO2 composites indicate an orthorhombic crystalline structure, suggesting an interaction between HCl-doped PANI and manganese dioxide. The crystallinity of the composite increases with the weight percentage of MnO2, as evidenced by the more pronounced peaks of MnO_2 in the XRD patterns. This confirms the successful formation of HCl-doped PANI- MnO_2 composites.

Temperature-dependent D.C. conductivity measurements were conducted using the KEITHLEY 2400 SourceMeter over 313K to 393K, a study not previously reported in the literature. The samples demonstrated good conductivity at elevated temperatures, with the highest conductivity observed for HCl-doped PANI-15wt% MnO2. The conductivity ranged from 2.25×10^{-4}S/cm to 8.5×10^{-7} S/cm for 0wt% MnO2 and from 9.03×10^{-4} S/cm to 1.05×10^{-5} S/cm for 15wt% MnO2 over the same temperature range. The electrical properties of polyaniline and HCl-doped PANI-MnO2 composites exhibited semiconducting behavior, with conductivity increasing with temperature due to enhanced charge transport efficiency between the polymer chains and the dopant. The activation energy decreased from 0.818 eV for pure PANI to 0.508 eV for HCl-doped PANI-15wt% MnO2, indicating improved charge carrier mobility with increasing MnO2 concentration.

The Seebeck coefficient measurements of HCl-doped PANI and its composites, also conducted in this study, were previously unreported. Using a nano-voltmeter 2182A, the voltage differences were observed, and the samples were prepared via the doctor-blade method with copper contacts. The Seebeck coefficient of HCl-doped PANI composites increased with both MnO2 concentration and temperature, reaching a maximum value of 52 mV/K at 373K, compared to 32 mV/K for HCl-doped PANI. Similarly, the current density of the samples increased with Temperature and MnO2 concentration, reaching a maximum of 17.57 mA/cm² from 3.57 mA/cm² at 373K. This enhancement is attributed to the wide band gap of the dopant, which elevates most carriers above the Fermi level. Consequently, the thermoelectric properties of HCl-doped PANI-MnO2 composites were significantly improved compared to HCl-doped PANI alone.

This research successfully demonstrates the synthesis and characterization of HCl-doped PANI and HCl-doped PANI-MnO2 composites, providing new insights into their temperature-dependent electrical conductivity and thermoelectric properties, previously unexplored.

Author Contribution

J.M., and **M.I.** were responsible for conceptualization, investigation, methodology, project administration, supervision, software, handled data curation, formal analysis, funding acquisition,

resources, validation, visualization, writing the original draft, and review and editing. **R.S.** and **S.T.** funding acquisition, resources, validation, visualization, original draft writing, and review and editing.

References:

1. Rowe, D. M. (1995). CRC Handbook of Thermoelectrics.
2. Tritt, T. M. (2001). Thermoelectric Materials: Principles, Structure, Properties, and Applications.
3. Snyder, G. J., & Toberer, E. S. (2008). Complex thermoelectric materials. Nature Materials, 7(2), 105-114.
4. Ramanathan, S. (2012). Thin Film Metal-Oxides: Fundamentals and Applications in Electronics and Energy.
5. Zhou, X., Jin, H., Yang, Y., & Wang, H. (2011). Conducting polymer nanostructures and their application in flexible thermoelectric devices. Materials Research Bulletin, 46(12), 2163-2166.
6. MacDiarmid, A. G., Chiang, J. C., Richter, A. F., & Epstein, A. J. (1985). Polyaniline: a new concept in conducting polymers. Synthetic Metals, 18(1-3), 285-290.
7. Chiang, J. C., & MacDiarmid, A. G. (1986). 'Polyaniline': Protonic acid doping of the emeraldine form to the metallic regime. Synthetic Metals, 13(1-3), 193-205.
8. Jang, S. Y., Oh, J. Y., & Bao, Z. (2014). Polyaniline for high-performance flexible thermoelectric materials. Journal of Materials Chemistry A, 2(16), 6327-6331.
9. Lu, Y., Yu, K., Liu, Z., Zhou, M., & Zhang, J. (2018). High-performance thermoelectric properties of polyaniline/multi-walled carbon nanotube nanocomposites. RSC Advances, 8(1), 575-581.
10. Whittingham, M. S. (2004). Lithium batteries and cathode materials. Chemical Reviews, 104(10), 4271-4302.
11. Bruce, P. G., Freunberger, S. A., Hardwick, L. J., & Tarascon, J. M. (2008). Li-O2 and Li-S batteries with high energy storage. Nature Materials, 11(1), 19-29.
12. Post, J. E. (1999). Manganese oxide minerals: crystal structures and economic and environmental significance. Proceedings of the National Academy of Sciences, 96(7), 3447-3454.
13. Zhou, G., Wang, D. W., Li, F., Zhang, L., Li, N., Wu, Z. S., & Cheng, H. M. (2010). Graphene-wrapped MnO2 nanocomposites for supercapacitor electrodes. Nano Research, 3(4), 224-233.
14. Zhao, Y., Liu, H., He, R., Bai, Y., Zhuang, X., & Yang, C. (2019). Polyaniline/manganese dioxide composite fibers for high-performance flexible supercapacitors. Journal of Power Sources, 413, 403-409.
15. Hoffman, R. M. (2002). In vivo studies. Proceedings of the National Academy of Sciences, 99(3), 1653-1654.
16. Lee, S. H., Lee, S., & Park, H. S. (2016). Highly durable and flexible thermoelectric films of polymer nanocomposites. Nature Communications, 7, 12011.
17. Chen, G., Wang, S., & Ren, Z. (2015). Recent developments in thermoelectric materials. International Materials Reviews, 60(4), 243-260.
18. Kim, F., Yang, S. J., & Park, C. R. (2013). Conducting polymer nanocomposites: progress and challenges. Chemical Society Reviews, 42(5), 1825-1838.

19. Cao, G. (2010). Nanostructures and Nanomaterials: Synthesis, Properties, and Applications.
20. Shakouri, A. (2011). Recent developments in semiconductor thermoelectric physics and materials. Annual Review of Materials Research, 41, 399-431.
21. Dresselhaus, M. S., Chen, G., Tang, M. Y., Yang, R. G., Lee, H., Wang, D. Z., & Ren, Z. F. (2007). New directions for low-dimensional thermoelectric materials. Advanced Materials, 19(8), 1043-1053.

Click here to access/download
Supporting Information
SI_24072024.docx
