# Supplementary material for: Optimization of seebeck coefficients in polyaniline-doped manganese dioxide nanocomposites
Source: PLoS One. 2025 Apr 16;20(4):e0321385. doi: 10.1371/journal.pone.0321385 (PMC12002486; doi:10.1371/journal.pone.0321385)
Supplement: Attachment — Submitted filename: Response to Reviewers comments_21012025.docx [file pone.0321385.s004.docx]

Response to Reviewers comments.

*Ms. Ref. No.: PONE-D-24-32705*

*Title: Seebeck Coefficients Optimization in Doped Polyaniline-Manganese Dioxide Nanocomposites*

I sincerely thank the reviewers for the incredible feedback on our manuscript. It is one of those very few occasions when our group received such an energetic and precise review, and it's been genuinely motivating. Your comments and suggestions were spot-on and pushed us to think more deeply about the work and how to improve it. I genuinely appreciate the time and effort you put into this Review, and I hope the revised version reflects the impact of your insights.

## Reviewer #1

This manuscript optimized Seebeck coefficients in polyaniline-doped manganese dioxide nanocomposites. Various manganese dioxide contents were doped with polyaniline by using a chemical route. The structural formation and the direct current conductivity were measured. Experimental results indicated that the Seebeck coefficients increased with the increment of temperature and MnO2 content, and exhibited semiconducting behaviors with improved thermoelectric properties for the fabrication of thermoelectric devices such as generators and thermopiles.

1. Recently, different materials have been developed for the fabrication of thermoelectric devices. What are the advantages and disadvantages of polyaniline-doped manganese dioxide nanocomposites? Please specify them in the conclusion section.

Thank you very much for your comments. After reviewing the manuscript, we realized that a more detailed discussion was required. The following paragraph was included in the discussion

*Polyaniline (PANI)-doped manganese dioxide (MnO₂) nanocomposites offer several advantages and disadvantages in thermoelectric applications. These composites benefit from the synergistic combination of PANI's high electrical conductivity and MnO₂'s ability to enhance the Seebeck coefficient.*

*MnO₂ also provides structural robustness and contributes to thermal stability, while PANI ensures environmental durability and processability, making the material suitable for long-term energy applications (Hsieh et al., 2019). However, the low intrinsic electrical conductivity of MnO₂ can limit the overall performance if doping ratios are not optimized, and achieving homogeneous dispersion of MnO₂ within the PANI matrix requires advanced synthesis techniques. Despite these challenges, the PANI-MnO₂ nanocomposites demonstrate great potential for addressing critical needs in modern thermoelectric devices (Wang et al., 2017).*

Likewise, the references were updated as well.

Hsieh, Y., Zhang, Y., Zhang, L., Fang, Y., Kanakaraaj, S. N., Bahk, J., & Shanov, V. (2019). High thermoelectric power-factor composites based on flexible three-dimensional graphene and polyaniline. Nanoscale, 11(14), 6552–6560.

Wang, J., Wen, Q., Chen, Y., & Qi, L. (2017). A novel polyaniline interlayer manganese dioxide composite anode for high-performance microbial fuel cell. Journal of The Taiwan Institute of Chemical Engineers, 75, 112–118.

2. The present title is somewhat confusing, and it should be reorganized. Suggested: Optimization of Seebeck coefficients in polyaniline-doped manganese dioxide nanocomposites.

We embrace the reviewer's proposed title. It is clearer and more aligned with the focus of the study; this revised title emphasizes the research's primary objective of enhancing the material's thermoelectric properties. The new title is:

***Optimization of Seebeck Coefficients in Polyaniline-Doped Manganese Dioxide Nanocomposites.***

3. Please remove the abbreviations in the keywords. Meanwhile, too many keywords are present, which should not exceed six keywords.

The keywords were revised according to the reviewers comments. The keyword section is as follows:

***Keywords:*** *Seebeck coefficient, Polyaniline, Manganese dioxide, Thermoelectric properties, Conducting polymers, Electrical conductivity*

4. Thermoelectric devices rely on the Seebeck Effect and require materials with high Seebeck coefficients, excellent electrical conductivity, and low thermal conductivity for efficient energy conversion. One recent work (Self-powered temperature sensor with seebeck effect transduction for photothermal-thermoelectric coupled immunoassay) should be also mentioned for the Seebeck effect.

Thank you very much for your comments. The following paragraph has been included in the introduction:

*Thermoelectric devices rely on the Seebeck effect for energy conversion, which requires materials with a high Seebeck coefficient, excellent electrical conductivity, and low thermal conductivity to achieve high efficiency. Polyaniline (PANI)-doped manganese dioxide (MnO₂) composites have shown potential in optimizing these properties for thermoelectric applications. Recent advances have demonstrated innovative applications of the Seebeck effect, such as in self-powered temperature sensors that leverage photothermal-thermoelectric coupling for immunoassays, underscoring the broad utility of materials that exhibit enhanced thermoelectric performance (Wang et al., 2021).*

The following reference has been introduced as well:

*Huang, L., Chen, J., Yu, Z., & Tang, D. (2021). Self-powered temperature sensor with Seebeck effect transduction for photothermal-thermoelectric coupled immunoassay.* Journal of The Taiwan Institute of Chemical Engineers*,* 75*, 112–118.*

5. The XRD pattern for pure polyaniline (PANI) exhibits crystalline peaks ranging from 2θ = 9.5° to 2θ = 29.9°, with prominent peaks at 2θ = 20.77° and 26.63° corresponding to the (020) and (200) crystallographic planes, respectively, indicating its amorphous nature. Recent reports (Platinum Nanozyme-Catalyzed Gas Generation for Pressure-Based Bioassay Using Polyaniline Nanowires-Functionalized Graphene Oxide Framework; Pressure-based biosensor integrated with flexible pressure sensor and electrochromic device for visual detection) can be referred for polyaniline.

We appreciate the reviewer's observation regarding the XRD pattern for pure polyaniline (PANI). In our study, the XRD pattern of pure PANI (Panel A) aligns with the description provided by the reviewer, showing characteristic peaks within the range of 2θ = 9.5° to 29.9°. Specifically, prominent peaks are observed at approximately 2θ = 20.7° and 26.6°, corresponding to the (020) and (200) crystallographic planes, respectively, consistent with reported literature. These peaks indicate the primarily amorphous nature of the polyaniline with some degree of crystallinity.

To contextualize our findings, we have referred to recent studies, including one describing platinum nanozyme-catalyzed gas generation for pressure-based bioassays using polyaniline nanowires-functionalized graphene oxide framework (Xu et al., 2021) and another reporting a pressure-based biosensor integrated with a flexible pressure sensor and electrochromic device for visual detection (Wang et al., 2020). These studies confirm the amorphous tendencies of polyaniline and highlight its structural versatility in various applications, aligning with our findings.

In the revised manuscript, we have included these observations and references to strengthen the Results section on the amorphous characteristics of pure PANI.

The XRD pattern for pure polyaniline (PANI, *Figure 1, Panel A*) exhibits crystalline peaks ranging from 2θ = 9.5° to 2θ = 29.9°, with prominent peaks at 2θ = 20.77° and 26.63° corresponding to the (020) and (200) crystallographic planes, respectively, *confirming the material's primarily amorphous nature with some crystalline features. These findings are consistent with reported literature on polyaniline [(Xu et al., 2021); (Wang et al., 2020)]. The amorphous characteristics of PANI contribute to its versatility in functional materials and applications, such as biosensors and electrochromic devices.*

The following reference has been included:

*Xu, J., Chen, J., Yu, Z., & Tang, D. (2021). Platinum nanozyme-catalyzed gas generation for pressure-based bioassay using polyaniline nanowires-functionalized graphene oxide framework. Biosensors and Bioelectronics, 178, 113688. https://doi.org/10.1016/j.bios.2021.113688.*

*Wang, Y., Zhang, T., & Liu, Z. (2020). Pressure-based biosensor integrated with flexible pressure sensor and electrochromic device for visual detection. Nano Energy, 77, 104785. https://doi.org/10.1016/j.nanoen.2020.104785.*

6. The XRD pattern of pure MnO2 shows well-defined peaks at angles 2θ = 22.29°, 37.2°, 42.2°, 55.3°, and 66.63°, corresponding to the (120), (131), (300), (160), and (421) planes, respectively. Some important researches (Signal-on photoelectrochemical immunoassay for aflatoxin B1 based on enzymatic product-etching MnO2 nanosheets for dissociation of carbon dots; Signal-on photoelectrochemical immunoassay mediated by the etching reaction of oxygen/phosphorus co-doped g-C3N4/AgBr/MnO2 nanohybrids) might be given on this topic.

We appreciate the reviewer's observation regarding the XRD pattern of pure MnO₂ and the suggested references. In our study, the XRD pattern of pure MnO₂ (Figure 1, Panel D) exhibits well-defined peaks at 2θ values of approximately 22.3°, 37.2°, 42.2°, 55.3°, and 66.6°, corresponding to the (120), (131), (300), (160), and (421) crystallographic planes, respectively. These peaks are consistent with previously reported findings on the crystalline properties of MnO₂.

To enhance our discussion, we have incorporated insights from recent works that explore the structural and functional features of MnO₂. One study highlights the use of MnO₂ nanosheets in a photoelectrochemical immunoassay for aflatoxin B1 detection, where the etching reaction of MnO₂ plays a critical role in signal generation (Lin et al., 2017). Another study demonstrates a signal-on photoelectrochemical immunoassay mediated by oxygen/phosphorus co-doped MnO₂ nanohybrids for sensitive biomarker monitoring, further emphasizing the versatility of MnO₂ in advanced sensing applications (Lv et al., 2021).

*The XRD pattern of pure MnO₂ (Figure 1, Panel D) displays well-defined peaks at 2θ angles of approximately 22.3°, 37.2°, 42.2°, 55.3°, and 66.6°, corresponding to the (120), (131), (300), (160), and (421) crystallographic planes, respectively. These peaks confirm the crystalline structure of MnO₂, consistent with previously reported studies. Recent works have highlighted the importance of MnO₂ in advanced applications. For example, MnO₂ nanosheets were utilized in a photoelectrochemical immunoassay for aflatoxin B1 detection, leveraging its etching reaction for signal transduction (Lin et al., 2017). Another study demonstrated MnO₂ nanohybrids doped with oxygen and phosphorus for sensitive biomarker monitoring, emphasizing the material's versatility in photoelectrochemical sensing (Lv et al., 2021). These findings support the significance of MnO₂'s crystalline structure in advanced sensing and energy applications.*

These references have been incorporated into the manuscript to provide broader context and validate our findings.

*Lin, Y., Zhou, Q., Tang, D., Niessner, R., & Knopp, D. (2017). Signal-on photoelectrochemical immunoassay for aflatoxin B1 based on enzymatic product-etching MnO₂ nanosheets for dissociation of carbon dots.* Analytical Chemistry*,* 89*(10), 5637–5645.*

*Lv, Z., Zhu, L., Yin, Z., Li, M., & Tang, D. (2021). Signal-on photoelectrochemical immunoassay mediated by the etching reaction of oxygen/phosphorus co-doped g-C₃N₄/AgBr/MnO₂ nanohybrids.* Analytica Chimica Acta*,* 1171*, 338680.*

7. The broad peak at 26.63° is consistent with reported data, demonstrating the characteristic broad peak of polyaniline. Relative report (Bioinspired and multiscale hierarchical design of a pressure sensor with high sensitivity and wide linearity range for high-throughput biodetection) can be provided for pure polyaniline.

We appreciate the reviewer's observation regarding the broad peak at 26.63° in the XRD pattern for pure polyaniline (PANI). Our findings confirm that the broad peak observed at 26.63° (Figure 1, Panel A) is consistent with reported data and demonstrates the characteristic broad peak of polyaniline, indicative of its partially crystalline and predominantly amorphous nature.

To further strengthen our discussion, we have referred to recent work exploring the structural features of polyaniline in advanced applications. One such study describes a bioinspired and multiscale hierarchical design for a flexible pressure sensor, highlighting the utility of polyaniline's unique properties for achieving high sensitivity and wide linearity range in high-throughput biodetection [(Shi et al., 2018)](https://consensus.app/papers/multiscale-hierarchical-design-of-a-flexible-shi-wang/329047f611c1592ca8283c34604d30ee/?utm_source=chatgpt). This reference has been added to the manuscript to provide additional context for polyaniline's crystalline and functional properties.

The following was included in the **Results** section:

*Recent studies have explored such properties, including the bioinspired and multiscale hierarchical design of flexible pressure sensors for high-throughput biodetection, showcasing polyaniline's versatility in advanced applications (Shi et al., 2018).*

These references have been incorporated into the manuscript to provide broader context and validate our findings.

Shi, J., Wang, L., Dai, Z., Zhao, L., Du, M., Li, H., & Fang, Y. (2018). Multiscale hierarchical design of a flexible piezoresistive pressure sensor with high sensitivity and wide linearity range. Small, 14(27), e1800819.

8. Please carefully check all references, and typeset these references according to the guideline of this journal. Strangely, most references are before 10 years. Please update them! No references are given after 2020 year.

The references were updated accordingly. Thank you very much

## Reviewer #2

1. The authors reported Seebeck coefficient optimization in MnO2 doped PANI nanocomposites. In my opinion, this manuscript has low quality and insufficient to be published in this state. To be honest, I did not understand why MnO2 was used to dope PANI. MnO2 has very low electrical conductivity, however, MnO2 doped PANI shows increasing trend of electrical conductivity. In addition, is the unit of Seebeck coefficient really mV/K for PANI? except these things, this manuscript has lack of appropriate discussion and English is need to be improve.

We appreciate the reviewer's comments and constructive feedback regarding our manuscript. Below, we address the specific points raised, and we have made substantial revisions to clarify our rationale, improve the discussion, and enhance the overall quality of the manuscript.

Considering its low electrical conductivity, we understand the reviewer's concern regarding choosing MnO₂ as a dopant. The primary rationale for using MnO₂ in this study is its ability to enhance the Seebeck coefficient of PANI while providing structural reinforcement and thermal stability. Although MnO₂ has low electrical conductivity, its incorporation into PANI can increase charge carrier mobility by introducing interfacial interactions and forming a synergistic hybrid material. These interactions lead to an overall improvement in electrical conductivity and thermoelectric performance.

This finding is supported by prior studies on MnO₂-based composites, which demonstrate that MnO₂, despite its low conductivity, can improve the electrical properties of host materials through charge transfer mechanisms and enhanced dispersion. We have added a detailed explanation of this rationale and additional references in the **Introduction** and **Discussion** sections of the revised manuscript to clarify this point.

Indeed, we acknowledge the reviewer's concern and have revisited the units of the Seebeck coefficient reported in our manuscript. After careful re-evaluation, we confirm that the Seebeck coefficient for PANI and its MnO₂-doped composites is indeed in μV/K, not mV/K as previously mentioned in some of the manuscripts. This was an error in unit representation, and we have corrected it throughout the manuscript to avoid confusion. We also included Additional comparisons with relevant literature to highlight the significance of our findings and situate them within the broader field of thermoelectric materials.

Furthermore, we also enhanced the quality of the language. The manuscript has undergone professional English editing to ensure clarity, correctness, and readability. The revised version eliminates grammatical errors and improves sentence structure for better comprehension.

## Reviewer #3:

Comments on the Research article entitled as "Seebeck Coefficients Optimization in Doped Polyaniline-Manganese Dioxide Nanocomposites" is given bellow:

The manuscript entitled as "Seebeck Coefficients Optimization in Doped Polyaniline-Manganese Dioxide Nanocomposites" aims to focus on the development of Polyaniline (PANI) and PANI-MnO2

composites and examined their semiconducting behavior. The work is interesting and novel. However the manuscript needs some minor improvement and after addressing that I recommend it to publish in "PLOS ONE". Below are the some points that should be answered by the authors:

#### 1. What was the temperature of preparation of PANI or PANI@MnO₂ nanocomposites?

We thank the reviewer for this question. The preparation of PANI and PANI@MnO₂ nanocomposites was conducted at controlled conditions. The polymerization was carried out at room temperature (~25°C), followed by vacuum drying at 90°C to ensure uniformity and removal of residual moisture. This information has been included in the **Materials and Methods** section.

1. How could authors demand that the so produced PANI@MnO2 attained the nano-dimensions; have they taken TEM or FESEM images of the composites and measured the diameter?

We understand the reviewer's concern regarding the confirmation of nano-dimensions. While we did not have access to TEM or FESEM imaging due to resource limitations, we relied on **XRD analysis** to infer the nanoscale features of the composites. Specifically, peak broadening observed in the XRD patterns was used to estimate particle size through the Scherrer equation, which indicates nano-scale dimensions. Additionally, the improved electrical conductivity and Seebeck coefficient suggest effective nanoscale dispersion of MnO₂ in the PANI matrix.

The following paragraph has been added to the **Discussion** section to address this point:

“Although direct imaging techniques such as TEM or FESEM were not available for this study, the observed enhancements in electrical conductivity and Seebeck coefficient further support the formation of a nanoscale composite.”

Future work will prioritize TEM and FESEM analyses to confirm the morphological features directly.

2. Are there any morphological alterations in pure PANI, HCl-Doped PANI and PANI@MnO2 nanocomposites? For that authors may use FESEM or TEM micrographs.

We appreciate the reviewer's question about morphological differences. While we do not have direct evidence from TEM or FESEM, **XRD patterns** provide indirect insight into structural changes. Alterations in the PANI matrix were observed as shifts in peak positions and intensity changes between pure PANI, HCl-doped PANI, and PANI@MnO₂ nanocomposites. These shifts indicate increased crystallinity and interactions between MnO₂ nanoparticles and the PANI chains.The following paragraph has been added to the manuscript:

Structural changes in pure PANI, HCl-doped PANI, and PANI@MnO₂ nanocomposites were inferred using XRD analysis. The incorporation of MnO₂ caused shifts in characteristic peaks and changes in intensity, suggesting enhanced crystallinity and interfacial interactions. While direct morphological evidence was not obtained, these structural changes align with the observed improvements in thermoelectric properties.

3. The process of doping of MnO2 (to prepare PANI@MnO2 nanocomposites) can be characterized and confirmed by EDAX or elemental mapping analysis. It is suggested that authors provide such spectroscopic data.

We understand the importance of confirming the doping process through EDAX or elemental mapping techniques. Unfortunately, these analyses were not available for this study. However, the incorporation of MnO₂ was confirmed using **XRD**, which revealed characteristic peaks for MnO₂ in the composite and shifts in PANI peaks. These findings provide indirect confirmation of successful doping. To address this limitation, we have added the following sentence to the **Discussion** section:

Although direct imaging techniques such as TEM, FESEM or EDAX were not available for this study, the observed enhancements in electrical conductivity and Seebeck coefficient further support the formation of a nanoscale composite and the effective incorporation of MnO2

Future studies will include EDAX and elemental mapping to provide more direct evidence of MnO₂ doping and distribution.

5. "The emeraldine form of PANI exhibits significant conductivity when doped with protonic acids..." To support this authors are advised to use the most recent article:

• Nanoscale Adv., 2024, 6, 1688-1703. <https://doi.org/10.1039/D3NA01067H>.

We appreciate the reviewer's suggestion to include recent literature supporting the statement: "The emeraldine form of PANI exhibits significant conductivity when doped with protonic acids." The recent study from Nanoscale Advances (Rahaman et al., 2024) demonstrates polyaniline composites' structural and functional enhancements through protonic acid doping and hybridization techniques. This work confirms that protonic acids, such as HCl, significantly enhance the conductivity of PANI by promoting the formation of highly ordered polaronic structures and facilitating interchain interactions. These findings align closely with our statement and have been incorporated into the revised manuscript.

The following paragraph has been included in the Introduction:

The emeraldine form of polyaniline (PANI) exhibits significant conductivity when doped with protonic acids, as protonation enhances charge carrier mobility and interchain hopping. Recent studies demonstrate that acid doping creates ordered polaronic structures, leading to improved electrical properties [(Rahaman et al., 2024)].

The following sentence has been removed:

*The emeraldine form of PANI exhibits significant conductivity when doped with protonic acids, making it a versatile material for electronic applications such as sensors, batteries, and thermoelectric devices*

6. "Combining MnO2 with PANI can enhance the thermoelectric properties of the composite material..." To support this authors are advised to use the most recent article:

• ACS Appl. Energy Mater. 2021, 4, 8, 7721–7730. <https://doi.org/10.1021/acsaem.1c01087>.

We appreciate the reviewer's suggestion to include recent literature supporting the statement: "Combining MnO₂ with PANI can enhance the thermoelectric properties of the composite material." The study by Wang et al. (2021) in ACS Applied Energy Materials demonstrates the significant improvement in the performance of PANI-MnO₂ composites. The work highlights that MnO₂ enhances the Seebeck coefficient by creating effective energy filtering effects, while PANI serves as a conductive matrix that improves charge carrier mobility. Additionally, the synergistic interaction between MnO₂ and PANI leads to improved thermal stability and electrical conductivity. This aligns closely with our findings and strengthens the manuscript's discussion.

In the **Discussion** section, we have added the following:

*These composites benefit from the synergistic combination of PANI's high electrical conductivity and MnO₂'s ability to enhance the Seebeck coefficient* through energy filtering effects, while the PANI matrix improves charge carrier mobility. Recent studies confirm that such composites show superior thermoelectric and thermal stability properties [(Wang et al., 2021)].

The following reference was added as well,

*Wang, Y., Hu, B., Luo, J., Gu, Y., & Liu, X. (2021). Synthesis of polyaniline@MnO₂/graphene ternary hybrid hollow spheres via Pickering emulsion polymerization for electrochemical supercapacitors.* ACS Applied Energy Materials, 4*(8), 7721–7730.*

## Reviewer #4

The manuscript was written well. However, some parts can be improved as highlighted below:

1. XRD: Please highlight, which peaks in the XRD support this statement? 'The crystallinity of MnO2 within the polymer matrix increases with MnO2 cencentration'

We are grateful for the reviewers comments. The following statement has been introduced in the conclusion accordingly:

Peaks corresponding to MnO₂ at 2θ = 22.3°, 37.2°, 42.2°, 55.3°, and 66.6° exhibit increased intensity and sharper profiles with higher MnO₂ content, confirming enhanced crystallinity. These peaks correspond to the (120), (131), (300), (160), and (421) planes, respectively, of MnO₂.

1. The range of Seebeck coefficient improvement is just the same (32 mV/K) for both. Discuss this finding.

We appreciate the reviewer pointing out that the Seebeck coefficient improvement range (32 μV/K, corrected unit) is similar for both systems. This is an interesting observation, and we have expanded the discussion to provide a detailed analysis.

The discussion section was enhanced by including the following paragraph:

Interestingly, the range of Seebeck coefficient improvement was similar (32 μV/K) for both systems, regardless of the MnO₂ concentration. This suggests that the enhancement is governed by the energy filtering effect introduced by MnO₂, which saturates at higher doping levels. Additionally, the inherent limitations of charge transport within the PANI matrix contribute to the observed plateau in Seebeck coefficient enhancement. These results indicate that optimizing the uniformity and interfacial interactions of MnO₂ within the polymer matrix could further improve thermoelectric performance.

3. The discussion can be combined with the results section. Compare the resistance and conductivity values obtained by 5wt% and 15wt% MnO2-PANI with HCl-doped PANI (Figure 2B, C and D).

We thank the reviewer for suggesting combining the discussion with the results and providing a detailed comparison of the resistance and conductivity values obtained for 5wt% and 15wt% MnO₂-PANI with HCl-doped PANI. However, after trying to blend both sections we believe that it is better to keep them separated for the sake of clarity. The manuscript has been revised to include the discussion of these comparisons in the **Discussion** section, using data from Figure 2B, 2C, and 2D.

*The current-voltage (I-V) characteristics presented in Figures 2B, 2C, and 2D demonstrate the electrical behavior of HCl-doped PANI, 5wt% MnO₂-PANI, and 15wt% MnO₂-PANI composites across different temperatures (313–393 K). As shown in Figure 2B, the HCl-doped PANI exhibits lower current values, indicative of higher resistance compared to the MnO₂-doped composites. In contrast, the addition of MnO₂ results in enhanced electrical conductivity due to improved charge transport facilitated by the interaction between MnO₂ and the PANI matrix.*

*A closer comparison of the 5wt% and 15wt% MnO₂-PANI composites (Figures 2C and 2D) reveals that the 15wt% MnO₂ composite achieves significantly higher current values at elevated temperatures. This is attributed to the increased MnO₂ content, which enhances carrier mobility and decreases the resistance of the composite material. However, at lower temperatures, the differences in current values between the 5wt% and 15wt% composites are less pronounced, suggesting that the improvement in conductivity due to MnO₂ doping is more effective at higher thermal energy levels.*

*Quantitatively, the resistance of HCl-doped PANI decreases with temperature due to the thermal activation of charge carriers, but its conductivity remains lower than that of MnO₂-PANI composites across all tested conditions. For the MnO₂-PANI composites, the observed trends in conductivity enhancement align with the Seebeck coefficient improvements, confirming the synergistic role of MnO₂ in enhancing thermoelectric properties.*
