# Supplementary material for: Optimization of seebeck coefficients in polyaniline-doped manganese dioxide nanocomposites
Source: PLoS One. 2025 Apr 16;20(4):e0321385. doi: 10.1371/journal.pone.0321385 (PMC12002486; doi:10.1371/journal.pone.0321385)
Supplement: Attachment — Submitted filename: Response to Reviewers comments_03032025.docx [file pone.0321385.s005.docx]

Response to Reviewers comments.

*Ms. Ref. No.: PONE-D-24-32705*

*Title: Optimization of Seebeck Coefficients in Polyaniline-Doped Manganese Dioxide Nanocomposites.*

We sincerely appreciate your valuable time and effort in reviewing our manuscript. Your insightful comments have significantly contributed to improving the quality of our work.

## Reviewer #1:

In this revised version, the authors have made a great effort to modify this manuscript according to the Reviewers’ comments. However, many concerns must be clarified and modified before being considered for publication in this journal. Too many references are wrong. Please carefully check throughout the text.

Ref. 8: “Huang, L., Chen, J., Yu, Z., & Tang, D. (2021). Self-powered temperature sensor with Seebeck effect transduction for photothermal-thermoelectric coupled immunoassay. Journal of The Taiwan Institute of Chemical Engineers, 75, 112–118” should be corrected to “Huang, L., Chen, J., Yu, Z., & Tang, D. (2020) Self-powered temperature sensor with seebeck effect transduction for photothermal-thermoelectric coupled immunoassay. Analytical Chemistry 92, 2809-2814.”
Ref. 27: “Xu, J., Chen, J., Yu, Z., & Tang, D. (2021). Platinum nanozyme-catalyzed gas generation for pressure-based bioassay using polyaniline nanowires-functionalized graphene oxide framework. Biosensors and Bioelectronics, 178, 113688” should be corrected to “Zeng, R., Luo, Z., Zhang, L., & Tang, D. (2018) Platinum nanozyme-catalyzed gas generation for pressure-based bioassay using polyaniline nanowires-functionalized graphene oxide framework. Analytical Chemistry 90, 12299-12306.”
Ref. 24: “Wang, Y., Zhang, T., & Liu, Z. (2020). Pressure-based biosensor integrated with flexible pressure sensor and electrochromic device for visual detection. Nano Energy, 77, 104785” should be corrected to “Yu, Z., Cai, G., Liu, X., & Tang, D. (2021) Pressure-based biosensor integrated with flexible pressure sensor and electrochromic device for visual detection. Analytical Chemistry 2021, 93: 2916-2925.”
Ref. 20: “Shi, J., Wang, L., Dai, Z., Zhao, L., Du, M., Li, H., & Fang, Y. (2018). Multiscale hierarchical design of a flexible piezoresistive pressure sensor with high sensitivity and wide linearity range. Small, 14(27), e1800819” should be corrected to “Huang, L., Zeng, R., Tang, D., & Cao, X. (2022). Bioinspired and multiscale hierarchical design of a pressure sensor with high sensitivity and wide linearity range for high-throughput biodetection. Nano Energy 99, 107376.”
Meanwhile, the corresponding references in the main text should be corrected according to the above-mentioned references.

Thank you for your time and detailed feedback. We carefully reviewed all references and have made the necessary corrections based on your suggestions. We also went through the entire reference list to ensure accuracy and consistency, correcting any other errors that may have been present.

We also wanted to mention that we encountered some glitches with the Mendeley reference manager, which affected other submissions and may have caused discrepancies in the original PDFs and the resulting reference list. To avoid further issues, we manually verified each reference this time. We hope that all errors have now been appropriately addressed. The whole reference list has been modified accordingly:

1. *Bruce, P. G., Freunberger, S. A., Hardwick, L. J., & Tarascon, J. M. (2012). Li-O2 and Li-S batteries with high energy storage. Nature Materials, 11(1), 19-29.*
2. *Cao, G. (2011). Nanostructures and nanomaterials: Synthesis, properties, and applications. (2nd ed.). World Scientific Publishing Company.*
3. *Cao, T., Shi, X., Zou, J., & Chen, Z. (2021). Advances in conducting polymer-based thermoelectric materials and devices. Microstructures, 1(1), 2021007.*
4. *Chen, G., Wang, S., & Ren, Z. (2015). Recent developments in thermoelectric materials. International Materials Reviews, 60(4), 243-260.*
5. *Chiang, J. C., & MacDiarmid, A. G. (1986). 'Polyaniline': Protonic acid doping of the emeraldine form to the metallic regime. Synthetic Metals, 13(1-3), 193-205.*
6. *Dresselhaus, M. S., Chen, G., Tang, M. Y., Yang, R. G., Lee, H., Wang, D. Z., & Ren, Z. F. (2007). New directions for low-dimensional thermoelectric materials. Advanced Materials, 19(8), 1043-1053.*
7. *Hoffman, R. M. (2002). In vivo studies. Proceedings of the National Academy of Sciences, 99(3), 1653-1654.*
8. *Hsieh, Y., Zhang, Y., Zhang, L., Fang, Y., Kanakaraaj, S. N., Bahk, J., & Shanov, V. (2019). High thermoelectric power-factor composites based on flexible three-dimensional graphene and polyaniline. Nanoscale, 11(14), 6552–6560.*
9. *Huang, L., Chen, J., Yu, Z., & Tang, D. (2020). Self-powered temperature sensor with Seebeck effect transduction for photothermal-thermoelectric coupled immunoassay. Analytical Chemistry, 92(5), 2809-2814.*
10. *Jang, S. Y., Oh, J. Y., & Bao, Z. (2014). Polyaniline for high-performance flexible thermoelectric materials. Journal of Materials Chemistry A, 2(16), 6327-6331.*
11. *Kim, F., Yang, S. J., & Park, C. R. (2013). Conducting polymer nanocomposites: Progress and challenges. Chemical Society Reviews, 42(5), 1825-1838.*
12. *Lee, S. H., Lee, S., & Park, H. S. (2016). Highly durable and flexible thermoelectric films of polymer nanocomposites. Nature Communications, 7, 12011.*
13. *Lin, Y., Zhou, Q., Tang, D., Niessner, R., & Knopp, D. (2017). Signal-on photoelectrochemical immunoassay for aflatoxin B1 based on enzymatic product-etching MnO₂ nanosheets for dissociation of carbon dots. Analytical Chemistry, 89(10), 5637–5645.*
14. *Lu, Y., Yu, K., Liu, Z., Zhou, M., & Zhang, J. (2018). High-performance thermoelectric properties of polyaniline/multi-walled carbon nanotube nanocomposites. RSC Advances, 8(1), 575-581.*
15. *Lv, Z., Zhu, L., Yin, Z., Li, M., & Tang, D. (2021). Signal-on photoelectrochemical immunoassay mediated by the etching reaction of oxygen/phosphorus co-doped g-C₃N₄/AgBr/MnO₂ nanohybrids. Analytica Chimica Acta, 1171, 338680.*
16. *MacDiarmid, A. G., Chiang, J. C., Richter, A. F., & Epstein, A. J. (1985). Polyaniline: A new concept in conducting polymers. Synthetic Metals, 18(1-3), 285-290.*
17. *Post, J. E. (1999). Manganese oxide minerals: Crystal structures and economic and environmental significance. Proceedings of the National Academy of Sciences, 96(7), 3447-3454.*
18. *Ramanathan, S. (2012). Thin film metal-oxides: Fundamentals and applications in electronics and energy. Springer.*
19. *Rowe, D. M. (1995). CRC Handbook of Thermoelectrics. CRC Press.*
20. *Shakouri, A. (2011). Recent developments in semiconductor thermoelectric physics and materials. Annual Review of Materials Research, 41, 399-431.*
21. *Huang, L., Zeng, R., Tang, D., & Cao, X. (2022). Bioinspired and multiscale hierarchical design of a pressure sensor with high sensitivity and wide linearity range for high-throughput biodetection. Nano Energy 99, 107376.*
22. *Snyder, G. J., & Toberer, E. S. (2008). Complex thermoelectric materials. Nature Materials, 7(2), 105-114.*
23. *Tritt, T. M. (2001). Thermoelectric materials: Principles, structure, properties, and applications. Springer.*
24. *Wang, J., Wen, Q., Chen, Y., & Qi, L. (2017). A novel polyaniline interlayer manganese dioxide composite anode for high-performance microbial fuel cell.* Journal of the Taiwan Institute of Chemical Engineers, 75*, 112–118.*
25. *Yu, Z., Cai, G., Liu, X., & Tang, D. (2021) Pressure-based biosensor integrated with flexible pressure sensor and electrochromic device for visual detection. Analytical Chemistry 2021, 93: 2916-2925.*
26. *Wang, Y., Hu, B., Luo, J., Gu, Y., & Liu, X. (2021). Synthesis of Polyaniline@MnO₂/Graphene Ternary Hybrid Hollow Spheres via Pickering Emulsion Polymerization for Electrochemical Supercapacitors. ACS Applied Energy Materials, 4(8), 7721–7730.*
27. *Whittingham, M. S. (2004). Lithium batteries and cathode materials. Chemical Reviews, 104(10), 4271-4302.*
28. *Zeng, R., Luo, Z., Zhang, L., & Tang, D. (2018). Platinum nanozyme-catalyzed gas generation for pressure-based bioassay using polyaniline nanowires-functionalized graphene oxide framework. Analytical Chemistry, 90, 12299-12306.*
29. *Zhao, Y., Liu, H., He, R., Bai, Y., Zhuang, X., & Yang, C. (2019). Polyaniline/manganese dioxide composite fibers for high-performance flexible supercapacitors. Journal of Power Sources, 413, 403-409. [Correction Needed]*
30. *Zhou, G., Wang, D. W., Li, F., Zhang, L., Li, N., Wu, Z. S., & Cheng, H. M. (2010). Graphene-wrapped MnO₂ nanocomposites for supercapacitor electrodes. Nano Research, 3(4), 224-233.*
